# Irrigation techniques used in spine surgery for surgical site infection prophylaxis: a systematic review and meta-analysis

**DOI:** 10.1186/s12891-022-05763-2

**Published:** 2022-08-26

**Authors:** Kabir A. Torres, Elliot Konrade, Jacob White, Mauro Costa M. Tavares Junior, Joshua T. Bunch, Douglas Burton, R. Sean Jackson, Jacob Birlingmair, Brandon B. Carlson

**Affiliations:** 1 Department of Surgery, The University of Missouri - Kansas City School of Medicine, 2301 Holmes Street, MO 64108 Kansas City, USA; 2 School of Medicine, The University of Kansas, Kansas City, USA; 3 Welch Medical Library, Johns Hopkins University, Baltimore, USA; 4 Department of Orthopedic Surgery, The University of Kansas Medical Center, 3901 Rainbow Blvd, MS 3017, Kansas City, KS Zip code: 66160 USA

**Keywords:** Spinal surgery, Infection prophylaxis, Surgical site infection, Surgical irrigation, Irrigation techniques, Complications

## Abstract

**Background:**

The greater likelihood of morbidity, mortality, length of hospital stays and poorer long-term outcomes as a result of surgical site infections secondary to spinal surgery makes prophylactic measures an imperative focus. Therefore, the aim of this review was to evaluate the available research related to the efficacy of different intraoperative irrigation techniques used in spinal surgery for surgical site infection (SSI) prophylaxis.

**Methods:**

We performed a comprehensive search using Ovid Medline, EMBASE, Web of Science and the Cochrane library pertaining to this topic. Our meta-analysis was conducted according to PRISMA guidelines. The inclusion criteria consist of spine surgeries with intraoperative use of any wound irrigation technique, comparison groups with a different intraoperative irrigation technique or no irrigation, SSI identified with bacterial cultures or clinically in the postoperative period, reported SSI rates. Data extracted from eligible studies included, but was not limited to, SSI rates, irrigation technique and control technique. Exclusion criteria consist of articles with no human subjects, reviews, meta-analyses and case control studies and no details about SSI identification or rates. Pooled risk ratios were calculated. A meta-analysis was performed with a forest plot to determine risk estimates’ heterogeneity with I^2^ index, Q-statistic, and *p* value under a random-effects model. Funnel plot was used to assess publication bias. All databases were last checked on January, 2022. PROBAST tool was used to assess both risk of bias and applicability concerns.

**Results:**

After reviewing 1494 titles and abstracts, 18 articles met inclusion criteria. They included three prospective randomized-controlled trials, 13 retrospective cohort studies, two prospective cohort studies. There were 54 (1.8%) cases of SSIs in the povidone-iodine irrigation group (*N* = 2944) compared to 159 (4.6%) in the control group (*N* = 3408). Using intraoperative povidone-iodine wound irrigation produced an absolute risk reduction of 2.8%. Overall risk ratio was 0.32 (95% CI 0.20–0.53, *p* < 0.00001). In a global analysis, study heterogeneity and synthesizing mostly retrospective data were primary limitations.

**Conclusion:**

The most evidence exists for povidone-iodine and has Level 2 evidence supporting SSI reduction during spinal surgery. Other antiseptic solutions such as dilute chlorhexidine lack published evidence in this patient population which limits the ability to draw conclusions related to its use in spinal surgery.

**Level of Evidence:**

II – Systematic Review with Meta-Analysis.

**Supplementary Information:**

The online version contains supplementary material available at 10.1186/s12891-022-05763-2.

## Background

Surgical site infections (SSI) are the most common complication after spinal surgery and are associated with increased morbidity, mortality, length of hospital stays and poorer long-term outcomes [1]. Reported SSI incidence is 4% [2], with even higher rates seen in implant-related surgery. These rates increase to 9.4% for instrumented spinal surgery for traumatic fractures and 19.2% in pediatric deformity surgery [3, 4]. Previous evidence showed that SSI accounted for 45.6% of readmissions among metastatic spine tumor patients [5].

A more recent study reports SSI rates ranging from 0.2% to 16.7% based on sub-stratified characteristics like patient risk factors, perioperative factors and pathology [6]. Risk factor mitigation along with appropriate preoperative, intraoperative and postoperative prophylactic measures are all methods for decreasing SSI during spinal surgeries. Evidence-based SSI prevention strategies should be implemented during spinal surgery and have tremendous opportunity to decrease morbidity, mortality and healthcare costs.

Intrawound decontamination prior to closure has become a unique point of interest for research. Present methods include direct application of antibiotic, most commonly intrawound, powdered vancomycin [7–9]. Intrawound vancomycin has been studied quite extensively in recent years. Intrawound antiseptic irrigation is another method of SSI prophylaxis that is often combined with intrawound vancomycin [10]. The majority of the research has focused on povidone-iodine, although concerns exist regarding its toxicity to osteocytes in spinal fusion procedures [11]. Other irrigation solutions have limited evidence validating their benefits and/or risks.

But there is a lack in the literature about the most effective and validated wound irrigation technique that could be used in spinal surgery to prevent SSI. Therefore, the purpose of this systematic review and meta-analysis is to evaluate the available research related to the efficacy of different intraoperative irrigation techniques used during spinal surgery for surgical site infection (SSI) prophylaxis.

We present the following article in accordance with the Preferred Reporting Items for Systematic Reviews and Meta-Analyses (PRISMA) reporting checklist.

## Methods

Our literature search strategy was to seek studies of elective spine surgery patients (population) treated with perioperative irrigation techniques (intervention) versus no or other irrigation technique (comparator) that assessed for surgical site infection (outcome). A comprehensive search was performed by a medical librarian from Ovid Medline, EMBASE, Web of Science and the Cochrane library with no publication date restrictions. The reviewers were not blinded to the journals, organizations, or author information. The following query or Medical Subject Headings (MeSH) terms included but were not limited to “spine”, “spinal diseases”, “spinal cord”, “therapeutic irrigation”, “lavage” and “wound lavage”. All search criteria terms used were developed with a medical librarian and can be found on Additional file 1: Appendix 1.

The inclusion criteria were as follow: (1) spine surgeries with intraoperative use of any wound irrigation technique, (2) comparison groups with a different intraoperative irrigation technique or no irrigation, (3) SSI identified with bacterial cultures or clinically in the postoperative period, (4) reported SSI rates. Only articles in English were considered.

Exclusion criteria: (1) articles with no human subjects, (2) reviews, meta-analyses and case control studies and (3) lack of details about SSI identification or rates.

**Fig. 1:**
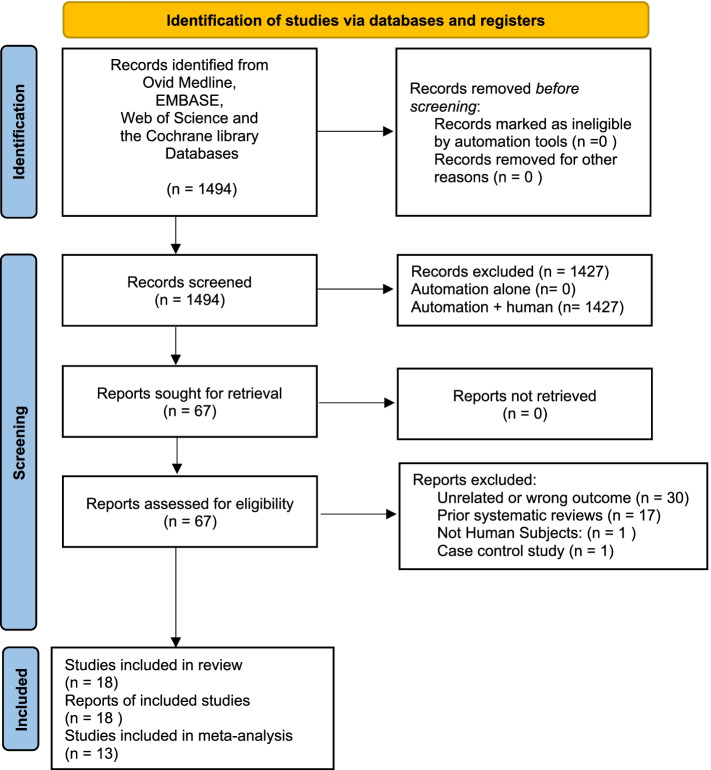
Flow diagram demonstrating article selection

Initial title and abstract screening was performed using Rayyan research tool from Qatar Computer Research Institute (QCRI—https://www.rayyan.ai/) [12] by two independent reviewers with a third independent reviewer to resolve any disagreement. After excluding irrelevant studies based on title and abstract, a second, screening process with full-text review was performed by both reviewers. Articles that were unrelated, unable to be retrieved in full text were excluded (Fig. 1).

We included retrospective cohort studies, prospective cohort studies and randomized controlled trials of humans that involved irrigation techniques application to spinal surgical wounds for postoperative SSI prophylaxis.

All databases were last queried on January 18, 2022. The protocol created for this review is detailed in Additional file 2: Appendix 2.

### Data extraction and outcome measures

For the meta-analysis, only studies related to povidone-iodine were analyzed to avoid increasing the heterogeneity and maintain high quality of evidence. Sub-analysis was performed in the povidone-iodine group including only RCT and prospective cohort studies to further increase the quality of evidence. The following variables were collected in a standardized Excel form: author list, publication years, sample sizes, irrigation technique with control group(s), rates of SSIs (superficial vs. deep when available). Exposure of interest was intraoperative irrigation technique to the surgical wound in spine surgeries. The primary outcome was the rate of SSIs reported in the irrigation technique and control groups. The accuracy of the data extraction was confirmed independently by 2 coauthors.

### Data syntheses

Descriptive statistics of the control versus experimental groups were combined and obtained using Microsoft Excel 365 (Microsoft Inc, Seattle, USA). Given the nonparametric nature of the data, chi-square statistics were performed between pooled data to identify any significant difference in observed and expected frequencies of SSIs. Pearson’s chi-square, risk ratio, 95% confidence intervals (CI), and *p* values were compiled. *P* < 0.05 was the standard of significance.

### Heterogeneity and publication bias

Outcome variable heterogeneity was evaluated using the Q statistic, Tau^2^, I^2^ index and *p* value under a random-effects model on a forest plot. The Cochrane Database Review Manager software was used to generate forest and funnel plots. A *P* value for the Q-statistic less than 0.1 suggests that significant data heterogeneity exists. Additionally, the I^2^ (range: 0%-100%) value establishes a quantitative degree of variation among included studies. The prediction model risk of bias assessment tool (PROBAST) was used for assessing the risk of bias and applicability by two reviewers working together.

## Results

### Literature search

The initial search using all search criteria yielded 1,494 articles. Initial screening of title and abstract excluded 1,427 articles leaving 67 articles for potential inclusion. Full text screening further eliminated 49 articles (articles that were unrelated, unable to be retrieved in full text, or previous systematic reviews) leaving 18 articles available for inclusion in the current study (Fig. 1).

The selected studies were three prospective randomized-controlled trials, 13 retrospective cohort studies, two prospective cohort studies. Publication years were as follows: 1998 (1), 2005 (2), 2006 (1), 2010 (1), 2013 (1), 2015 (1), 2016 (1), 2017 (2), 2018 (2), 2019 (2), 2020 (3) and 2021 (1) (Table 1).

**Table 1:** Summary of all studies

Reference	Year	N	Study Design	Patient Population	Primary Spine Diagnosis	Spine Location	Irrigation Technique	Volume of Irrigation Fluid	Total Infection Rate	Deep Infection Rate	Superficial Infection Rate
De Luna et al. [13]	2017	50	Prospective cohort	Adult	Scoliosis	Varied	Povidone-Iodine 0.3%	2 L	0% vs 12% (Post vs Pre)	N/A	N/A
Fei et al. [14]	2017	160	Prospective cohort	Adult	Prolapsed lumbar intervertebral discs	Lumbar	Povidone-Iodine 0.1%	200 mL	0% vs 20% (Iodine vs Saline, Muscle layer)	N/A	N/A
Cheng et al. [15]	2005	417	RCT	Adult	Varied	Varied	Povidone-Iodine 0.35%	5 mL diluted	0% vs 3.4% (Post vs. Pre)	0% vs 2.9% (Post vs. Pre)	0% vs 0.5% (Post vs. Pre)
Chang et al. [16]	2006	435	RCT	Adult	Lumbosacral segmental instability	Lumbosacral	Povidone-Iodine 0.35%	N/A	0% vs 4.8%	N/A	N/A
Sigari et al. [17]	2020	936	RCT	Adult	Scoliosis or other degenerative disease	Varied	Povidone-Iodine 3.0% soaked for 2 min	N/A	1.1% (Study) vs 4.5% (Control)	0.42% (Study) vs 1.9% (Control)	0.64% (Study) vs 2.6% (Control)
Savitz et al. [18]	1998	50	Retrospective cohort	Adult	N/A	N/A	50,000 units polymyxin and 50,000 units of bacitracin	1L saline	N/A	N/A	N/A
Mastronardi [19]	2005	1167	Retrospective cohort	Adult	Disc herniation	Lumbar	Rifamicin	N/A	0.63% (Study) vs 0.66% (Control)	N/A	N/A
Watanabe et al. [20]	2010	223	Retrospective cohort	Pediatric and Adult	Varied	Varied	Saline	N/A	6,3%	N/A	N/A
Kaliaperumal [21]	2013	3063	Retrospective cohort	Adult	Microdiscectomies	Lumbar	Savlon (chlorhexdine)	N/A	0.18% (Saline) vs 0.09% (Savlon)	N/A	N/A
Tomov et al. [22]	2015	2425	Retrospective cohort	Adult	Varied	Varied	Povidone-Iodine 0.3%	N/A	1.3% vs 2.4% (Post vs Pre)	N/A	N/A
van Herwijnen [23]	2016	118	Retrospective cohort	Pediatric	Scoliosis	Varied	80 mg Gentamicin diluted in saline	1L	26.7% (Gentamicin) vs 7.0% (PVP-I) vs 6.2% (PVP-I + Vanc)	20.0% (Gentamicin) vs 4.2%% (PVP-I) vs 3.1% (PVP-I + Vanc)	6.7% (Gentamicin) vs 2.8% (PVP-I) vs 3.1% (PVP-I + Vanc)
Yamada et al. [24]	2018	1042	Retrospective cohort	Adult	N/A	Varied	Povidone-Iodine 0.38%	20 mL diluted in 500 mL saline	0.7% vs 3.8% (Post vs Pre)	N/A	N/A
Karaarslan [25]	2018	166	Retrospective cohort	Adult	Spinal stenosis and spondylolisthesis	Lumbar	3 mL Rifampicin diluted with 5 mL saline	8 mL	1.2% (Rifampicin) vs 2.5% (Saline)	N/A	N/A
Lemans et al. [26]	2019	853	Retrospective cohort	Adult	N/A	Varied	Povidone-Iodine 0.013%	500 mL at 1.3 g/L	N/A	9.7% vs 9.7% (Post vs. Pre)	0.9% vs 5.1% (Post vs. Pre)
Onishi et al. [27]	2019	323	Retrospective cohort	Adult	N/A	Varied	Povidone-Iodine 1.0% pooled every 1.5 h	N/A	1.7% (Study) vs 3.4% (Control)	0.0% (Study) vs 2.7% (Control)	1.7% (Study) vs 0.7% (Control)
Tipper et al. [28]	2020	414	Retrospective cohort	Pediatric	Scoliosis	Varied	Saline	N/A	2,2%	N/A	N/A
Chen et al. [29]	2020	2626	Retrospective cohort	Adult	Spinal canal stenosis, disc herniation, spondylolisthesis, and/or degenerative scoliosis;	Thoracic/Lumbar	3% Hydrogen Peroxide	50 mL	1.4% (Hydrogen Peroxide) vs 2.4% (Saline)	0.2% (Hydrogen Peroxide) vs 1.1% (Saline)	1.2% (Hydrogen Peroxide) vs 1.3% (Saline)
Carballo Cuello et al. [30]	2021	278	Retrospective cohort	Adult	N/A	Lumbar	Povidone-Iodine 0.35%	35 ml of sterile 10% PVP-I in 1L of 0.9% saline	6.7% (saline) vs 0.7% (PVP-I	N/A	N/A

*N* Number, *N/A* Not applicable, *RCT* Randomized clinical trial

**Fig. 2:**
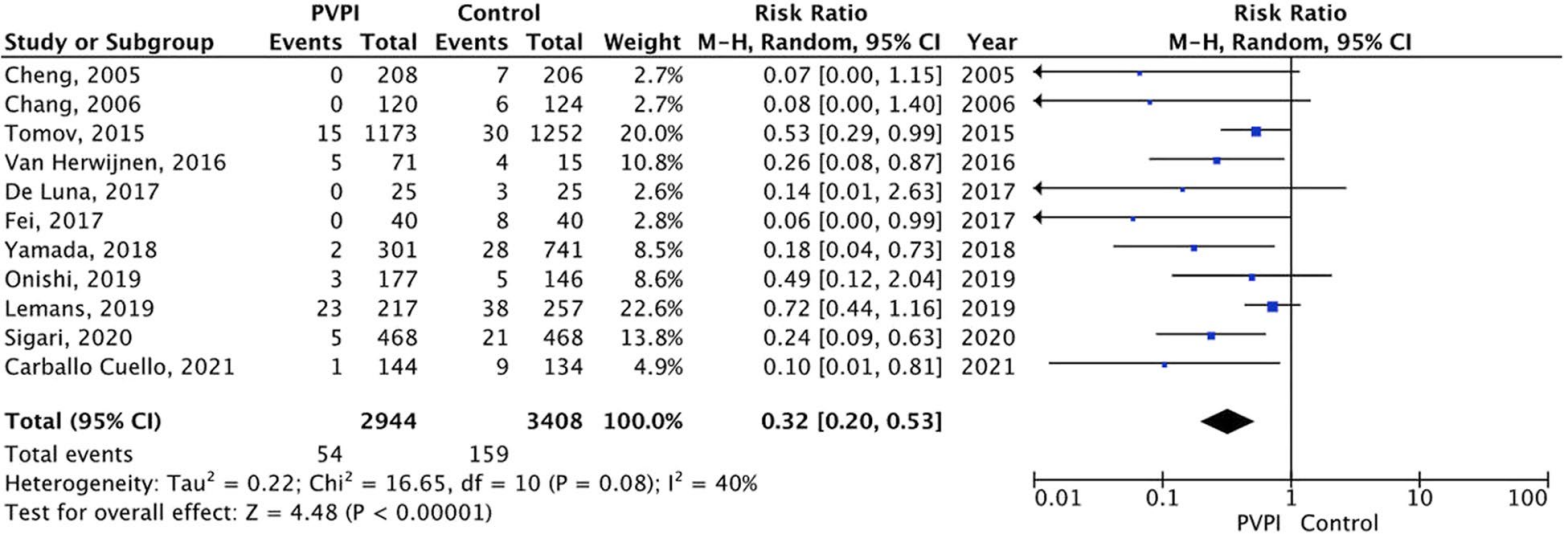
Comparison between intraoperative wound irrigation with povidone-iodine versus controls in their association with SSIs. Legends: CI, confidence interval; M-H, Mantel–Haenszel; PVPI, povidone-iodine

**Fig. 3:**
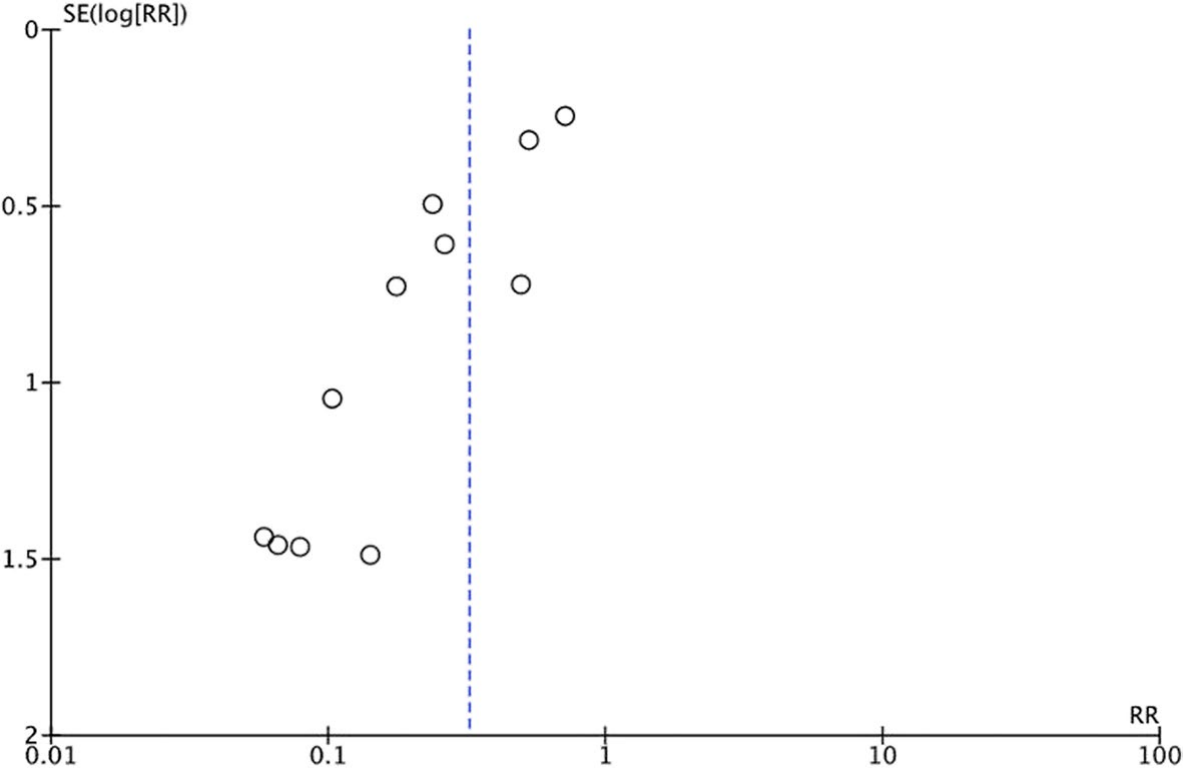
Funnel plot of the association between estimated effect size of each povidone-iodine study and standard error. Legends: RR, risk ratio; SE, standard error

When analyzing all studies that used povidone-iodine wound irrigation technique, there were 54 (1.8%) cases of SSIs in the povidone-iodine irrigation group (*N* = 2944) compared to 159 (4.6%) in the control group (*N* = 3408). Using intraoperative povidone-iodine wound irrigation produced an absolute risk reduction of 2.8%. Overall risk ratio was 0.32 (95% CI 0.20–0.53, *p* < 0.00001). Significant heterogeneity was present across all povidone-iodine studies (*p* = 0.08, I^2^ 40% (Fig. 2). The funnel plot found evidence of publication bias favoring the use of povidone-iodine in reducing overall SSI rates compared to controls (Fig. 3).

**Fig. 4:**
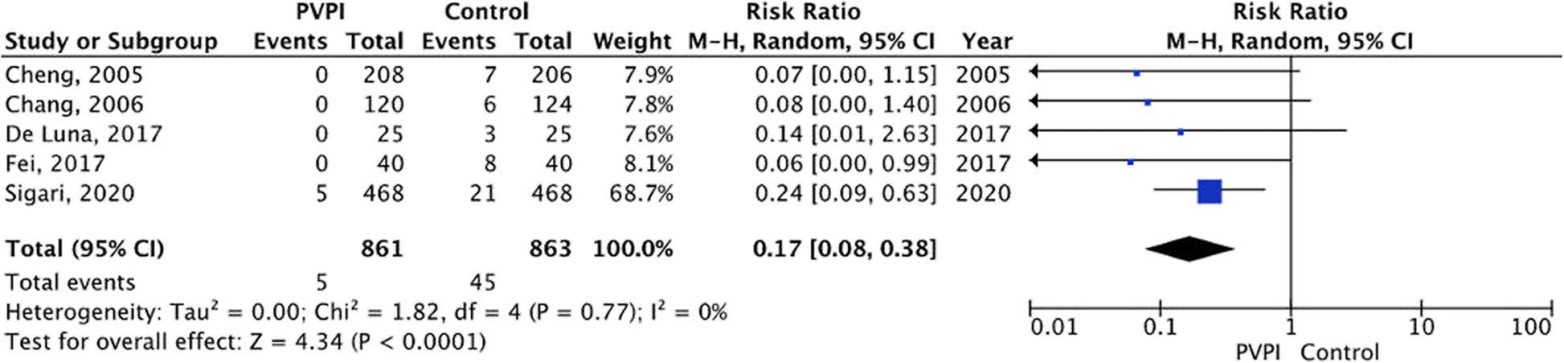
Comparison between povidone-iodine (only RCT and prospective cohort studies) versus controls in their association with SSIs. Legends: PVPI, povidone-iodine; RCT, randomized clinical trials; CI, confidence interval; M-H, Mantel–Haenszel

Figure 4 shows the forest plot sub-analysis of povidone-iodine irrigation technique including only RCT and prospective cohort studies.

### Study quality and risk of bias

The concerns of bias in the studies were primarily related to study participation (e.g., concerns about sampling/recruiting), lack of control for confounding, and follow-up losses. The PROBAST tool was used for assessing articles. It is organized into 4 domains: participants, predictors, outcome, and analysis [31]. All 20 studies included in this systematic review were assessed using this tool (Table 2).

**Table 2:** PROBAST Results

Study	ROB				Applicability			Overall	
	**Participants Predictors Outcome Analysis**				**Participants Predictors Outcome**			**ROB Applicability**	
**Watanabe et al. **[20]	+	+	?	+	+	+	+	?	+
**Tipper et al. **[28]	+	+	?	+	+	+	?	?	?
**Cheng et al. **[15]	+	+	?	+	+	+	+	?	+
**Chang et al. **[16]	+	+	?	+	+	+	?	?	?
**Tomov et al. **[22]	+	+	+	+	+	+	?	+	?
**De Luna et al. **[13]	+	+	+	+	+	+	?	+	?
**Fei et al. **[14]	?	+	+	+	+	+	?	?	?
**Yamada et al. **[24]	+	?	?	+	+	?	?	-	?
**Lemans et al. **[26]	+	+	+	+	+	+	?	+	?
**Onishi et al. **[27]	?	+	+	+	+	+	+	?	+
**Sigari et al. **[17]	+	+	+	+	+	+	+	+	+
**Savitz et al. **[18]	+	+	?	+	+	?	?	?	-
**Mastronardi **[19]	+	?	-	+	+	+	-	-	-
**van Herwijnen **[23]	+	?	+	+	+	+	+	?	+
**Karaarslan **[25]	-	+	+	+	+	+	?	-	?
**Chen et al. **[29]	+	+	+	+	+	+	?	+	?
**Kaliaperumal **[21]	+	+	?	+	+	+	?	?	?
**Carballo Cuello et al. **[30]	+	?	?	+	+	+	+	?	+

*PROBAST* Prediction model Risk Of Bias Assessment tool, *ROB* Risk of bias

+ indicates low ROB/low concern regarding applicability;

- indicates high ROB/high concern regarding applicability;

? indicates unclear ROB/unclear concern regarding applicability

### Saline

Normal saline is commonly used for surgical site irrigation. There are few studies that explicitly look at saline irrigation in spine surgery; however, Watanabe et al. demonstrated that using a sufficient amount of saline for irrigation (mean > 2000 mL/hour) showed a strong association with surgical site infection reduction (OR 0.08, 95% CI 0.01–0.61; *p* = 0.015) [20]. Only one other study examined saline delivered via pulsed lavage for a total of 3L [28]. This was in combination with a large protocol that was implemented in a single-center study and therefore individual statistical inferences about the impact of irrigation liquid and technique alone are unable to be drawn.

### Povidone-iodine

Povidone-iodine is an antiseptic solution consisting of polyvinylpyrrolidone with water, iodide and 1% available iodine with bactericidal ability against a large array of pathogens [32]. The advent of povidone-iodine (PVP-I) solutions for surgical site infections was widely accepted [33] even though the volume of research regarding its efficacy and risks is varied between surgical subspecialties. Its use in spine surgery is the most researched irrigation technique in the literature with the two most well-known and commonly cited studies being from Cheng et al. (2005) and Chang et al. (2006). These two single-blind RCTs compared normal saline to 0.35% PVP-I solutions. In their study group they used 0.35% concentrated PVP-I solution that stayed in the wound for 3 min, followed by a second washout with 2L of normal saline to remove the PVP-I solution. Both demonstrated a statistically significant decrease in deep (0% vs 2.9%, *p* = 0.015, Cheng; 0% vs 4.8%, *p* = 0.029, Chang) and total infection rates (0% vs 3.4%, *p* = 0.007, Cheng; 0% vs 4.8%, *p* = 0.029; Chang) [15, 16].

Almost a decade later, several studies examined the efficacy of PVP-I combined with other SSI prophylaxis methods. In 2015, Tomov et al. implemented a protocol consisting of a combination of 0.3% PVP-I for surgical site irrigation and 1 g of vancomycin powder [22]. The protocol was implemented over a four-year period split equally between a pre-intervention period and post-intervention period with the primary outcome being suspicion of infection requiring subsequent irrigation and debridement. The study’s results demonstrated an SSI reduction from 2.4% to 1.3% (*p* = 0.0287) [22]. This is the first published study examining the combination of PVP-I irrigation with vancomycin powder in spinal wounds. Unfortunately, the study did not evaluate PVP-I alone versus saline alone or vancomycin powder alone.

In 2017, De Luna et al. performed a prospective cohort study with a group of 50 consecutive adult patients over a two-year period who underwent spinal deformity surgery. Group A received low-pressure irrigation with PVP-I diluted to a 3% concentration in 2L of saline over a time period between 5 and 10 min followed by a 1L wash out with saline solution through a pulsed irrigation device. Group B received low-pressure irrigation with 2L saline solution over a time period between 5 and 10 min. Cultures were taken directly from the surgical site before and after irrigation. From samples before irrigation in both groups, contamination was experienced in 4 of 25 patients, but no patient developed clinical signs of infection in group A. On the other hand, three patients in Group B developed SSIs. The study suggested that pulsed irrigation with dilute PVP-I and saline may reduce infections but due to an underpowered sample size, statistical analyses were not able to be performed [13].

In the same year, Fei et al. retrospectively studied saline lavage, pulsed lavage, closed drainage and iodine lavage in 160 patients undergoing posterior lumbar interbody fusion over 2 years. Patients were evenly and randomly divided into each group. After irrigation, specimens were obtained via cotton swab from the posterior back muscles and intradiscal space. They found the pulsed lavage, closed drainage and iodine lavage showed a lower infection rate at the muscle when compared with the saline lavage group (*p* < 0.001). There was no significant difference in infection rate of intervertebral discs among the groups (*p* = 0.104) [14].

A retrospective observational study in 2018 using prospectively collected data was performed by Yamada et al. This utilized an SSI prevention care bundle in instrumented spinal surgery. The bundle included using diluted PVP-I wound irrigation along with preoperative additional IV vancomycin prophylaxis and preoperative nasal and body decontamination. The study reported a significant decrease in SSI rate from 3.8% to 0.7% (*p* < 0.01) along with a significant protective effect of the bundle observed through multivariate analysis (adjusted odds ratio 0.18, 95% confidence interval: 0.04–0.77, *p* = 0.02) [24].

In 2019, Lemans et al. conducted a retrospective cohort study to evaluate the efficacy of intrawound PVP-I or vancomycin powder in reducing deep and superficial SSI in instrumented spinal surgery. The prophylaxis group received 500 mL of PVP-I irrigation at 1.3 g/L concentration for 2 min followed by a wash out with saline. They reported no significant difference (9.7% to 9.7%, RR: 1.00, 95% CI 0.57–1.73) in the deep SSI incidence between the control group (saline only) and the PVP-I irrigation group. However, there was a significant reduction in superficial infections in the PVP-I group (5.1% to 0.9%, RR: 0.18, 95% CI 0.04–0.80) [26].

In a retrospective cohort study from Onishi et al. from 2019, a specific protocol outlining the pooling time of PVP-I and irrigation was examined. The study group consisted of 177 patients who received normal saline irrigation after 90 s of 1% PVP-I pooling every 1.5 h. The control group consisted of 146 patients who received routine saline irrigation every 1.5 h. Their primary result was a significant decrease in deep SSI rate between groups (*p* = 0.027), however, overall (superficial + deep) SSI rates were not different. Their findings suggest a beneficial effect of adding PVP-I to the irrigation solution in preventing deep SSI’s [27].

In 2020, Sigari et al. reported on 936 spinal fusion surgery patients who were randomized to receive irrigation with either 3% PVP-I solution followed by normal saline for a maximum of two minutes (study group) or normal saline (control group). They did not find significant decreases in deep and superficial infections when analyzed individually, but did report the overall infection rate (deep + superficial) was reduced in the PVP-I group (4.8% to 1.1%, *p* = 0.032) [17].

The most recent study was a retrospective report published in 2021 by Carballo Cuello et al. and compared consecutive patients who underwent elective posterior lumbar instrumentation and fusion in two spinal surgery cohorts. The first group was 134 patients irrigated before arthrodesis and closure with 1L of 0.9% normal saline solution; the second group was 144 patients irrigated with 35 mL of sterile 10% PVP-I. The authors found a 6.7% SSI rate in the normal saline group versus a 0.7% SSI rate in the PVP-I group (*p* = 0.008). The PVP-I solution had a relative risk for SSI of 0.093 (*p* = 0.008) and an adjusted odds ratio of 0.113 ( *p* = 0.05) [30].

### Antibiotic

Five studies defining SSI rates with antibiotic solution prophylaxis were identified. This is distinct from the antibiotic powders commonly used intraoperatively such as intrawound vancomycin [34]. In 1998, Savitz et al. looked at using a combination of diluted bacitracin and polymyxin in saline as a method of irrigation for SSI prophylaxis. The study consisted of 50 consecutive procedures with varying indications. Each liter of saline contained 50,000 units each of polymyxin and bacitracin. At the end of the study, no wound infections were documented in the 50 consecutive patients [18]. A 1979 study reported a 5-year eradication of operative infections during neurological surgery using a combination intramuscular gentamycin or tobramycin, intravenous vancomycin, and streptomycin irrigation [35]. Savitz et al. demonstrated that their combination of bacitracin and polymyxin was a suitable replacement for streptomycin as part of the antibiotic prophylaxis for cervical and lumbar spinal surgery [18].

Mastronardi et al. retrospectively compared saline plus rifamycin in a group of 450 patients against saline only in a group of 717 patients in 2005. The primary outcome was spondylodiscitis. No significant difference was found between groups [19]. An aminoglycoside, gentamicin, was studied by van Herwijnen et al. in 2015 in the pediatric population. Regimen A consisted of 6L of saline irrigation followed by 1L of saline mixed with 80 mg of gentamicin. Regimen B consisted of 3L of saline irrigation followed by 1L of 1% PVP-I soaking in the wound for 3 min before a second wash with 3L of saline. Regimen C was the same as Regimen B but irrigation was followed by 1 g vancomycin powder application. The results showed a 26.7% infection rate for Regimen A, 7.0% for Regimen B and 6.3% for Regimen C, although the differences were not statistically significant due to study limitations (small sample size, short follow up period, low risk patients studied) [23].

In 2018, Karaarslan et al. performed a retrospective review of 166 consecutive instrumented surgeries for lumbar spinal stenosis and spondylolisthesis. The study group consisted of rifampicin-washed implants and irrigation with dilute 3 mL rifampicin solution in 5 mL of normal saline. The control group had no rifampicin application or saline irrigation. There were no differences in infection rates between groups and the authors suggested a larger series was needed to verify the results [25].

### Hydrogen peroxide

In 2020, Chen et al. investigated the safety and efficacy of hydrogen peroxide with a primary outcome being reduction in SSI. In their retrospective study, 2626 posterior lumbar interbody fusion patients were included. The control group received 1L saline irrigation prior to closure and the study group received 50 mL of 3% hydrogen peroxide solution soaked for 30 s prior to 1L saline irrigation. The total SSI rates were not statistically different at 2.4% versus 1.4% in control and study groups, respectively (*p* = 0.068). They did report a significant decrease in deep wound infections reducing from 1.1% to 0.2% (*p* = 0.006) [29].

### Chlorhexidine

Only one study was identified studying the use of chlorhexidine irrigation as a prophylactic irrigation in spinal surgery. Kaliaperumal et al. studied an antiseptic composed of a cetrimide and chlorhexidine gluconate commonly sold as an antiseptic cream in the United Kingdom (Savlon; Novartis Consumer Health UK Limited, Surrey, UK). The primary outcome was spondylodiscitis. It was unclear how the antiseptic was diluted in saline, but the study demonstrated that irrigation with the solution resulted in a decrease in SSI rates from 0.18% to 0.09% when compared to saline irrigation alone [21]. This, however, was not deemed statistically significant as no statistical tests appeared to have been performed. There clearly is a paucity of literature related to chlorhexidine solutions used as irrigation in spinal surgery.

## Discussion

This review demonstrates that the ideal method and solution for surgical site irrigation during spine surgery remains unclear. There is more research regarding PVP-I than other methods, but in the majority of studies, there are several limitations for clear recommendation due to insufficient statistical power, ambiguous inclusion criteria or outcomes, mixture of different techniques, no clear standardization of the solution, etc. Toxicity to fibroblasts and the theoretical risk of causing pseudoarthrosis was a concern in the past, but recent studies have suggested PVP-I is safe and may not create tissue toxicity [15, 23].

A gap exists in this area of research and further studies should be implemented to better compare different irrigation techniques, volumes, and newer antiseptic solutions that have not been studied as extensively. Numerous studies demonstrate antiseptic irrigation has advantages compared to saline alone [13, 14, 26, 27, 36].

Evidence supporting antibiotic irrigation is limited to solutions combining bacitracin and polymyxin [18]; however, this evidence is likely outdated and more research into this method of irrigation prophylaxis needs to be conducted. Another concern about antibiotic irrigation would be the possibility of increasing the antimicrobial resistance worldwide [37]. A prior study in 1972, from Leonard Malis at Mount Sinai Hospital, excluded from this review because of the population studied (neurosurgical instead of spinal surgery cases), demonstrated a zero-infection rate among 1,732 consecutive neurosurgical operations. This was a result of a prophylactic bundle consisting of vancomycin 1 g IV, gentamicin 80 mg intramuscularly at the beginning of each case and streptomycin 50 mg in each liter of saline irrigating solution [35]. This so-called “Malis regimen” was proven efficacious for neurosurgical applications by several studies [36, 38–41] and was used at several institutions until streptomycin was no longer available. As to why antibiotic prophylaxis has not been investigated further with results such as this is unclear, but it could be an area of future study to determine the safety and efficacy of more recently developed antibiotics.

Based on World Health Organization global guidelines for the prevention of SSI, consideration to the irrigation of the incisional wound with PVPI solution should be made, particularly in clean and clean-contaminated wounds, but antibiotic incisional wound irrigation should not be used to prevent SSI [42]. This fact is in agreement with our meta-analysis results in which povidone-iodine wound irrigation produced an absolute risk reduction of 2.8% and overall risk ratio was 0.32 (95% CI 0.20–0.53, *p* < 0.00001).

This review identified very little research published related to chlorhexidine solutions. In other surgical specialties such as cardiac, orthopedic, and gynecological surgery, chlorhexidine has been used with minimal evidence of tissue toxicity [43, 44]. However, in the case of vaginal preparation PVP-I was shown to be more effective [44]. Also, it has been demonstrated that surgeons should be hesitant in mixing chlorhexidine with other antiseptics as precipitate can form and lead to complications [45]. Although chlorhexidine gluconate (CHG) has been available as topical antiseptic for over 50 years, there are newer surgical wound irrigation products composed of 0.05% CHG commercially available. The safe use of this solution has been reported in general surgery, cardiothoracic, orthopedic and obstetrical procedures and was reported with a capacity to reduce > 5-log the burden of health care-associated pathogens at the surgical site after 1 min exposure [46]. This topic deserves future prospective studies to determine SSI prevention in various surgical populations.

The strengths of this study include narrowing the scope to purely spinal surgery irrigation techniques, using a standardized PRISMA systematic review and meta-analysis format and using standardized software for article evaluation and article review performed by two independent reviewers. This review also has several limitations. Articles with titles or abstracts that did not clearly meet our inclusion criteria but did actually include spinal surgeries could have inadvertently been excluded. The small number of studies in each category and lack of standardized results limits our ability to draw definitive conclusions or perform a more powerful meta-analysis. In addition, we limited our review to irrigation techniques, however, many studies now include additional methods for SSI reduction including preoperative skin cleansing and intrawound antibiotic powders. Also, it is important to mention the lack of reporting additional details about demographic characteristics, journal or other publications details. Given the multiple confounding variables, it is difficult to make robust conclusions about the irrigation methods in isolation.

## Conclusion

The most evidence exists for povidone-iodine and has Level 2 evidence supporting SSI reduction during spinal surgery. Other antiseptic solutions such as dilute chlorhexidine lack published evidence in this patient population which limits the ability to draw conclusions related to its use in spinal surgery. This review demonstrates the need for further investigation through well designed studies assessing wound irrigation solutions and techniques in the setting of spine surgery and their effect on reduction of surgical site infections and possible related complications.

## Supplementary Information

**Additional file 1: Appendix 1.** Database Search Strategies.

**Additional file 2: Appendix 2.** Protocol.

## Data Availability

Datasets generated during and/or analyzed during the current study are available from the corresponding author on reasonable request.
