# Supplementary material for: Irrigation techniques used in spine surgery for surgical site infection prophylaxis: a systematic review and meta-analysis
Source: BMC Musculoskelet Disord. 2022 Aug 26;23:813. doi: 10.1186/s12891-022-05763-2 (PMC9414142; doi:10.1186/s12891-022-05763-2)
Supplement: Supplementary file 1 — Additional file 1: Appendix 1. Database Search Strategies. [file 12891_2022_5763_MOESM1_ESM.doc]

**Appendix I.**
