# Supplementary material for: Irrigation techniques used in spine surgery for surgical site infection prophylaxis: a systematic review and meta-analysis
Source: BMC Musculoskelet Disord. 2022 Aug 26;23:813. doi: 10.1186/s12891-022-05763-2 (PMC9414142; doi:10.1186/s12891-022-05763-2)
Supplement: Supplementary file 2 — Additional file 2: Appendix 2. Protocol. [file 12891_2022_5763_MOESM2_ESM.doc]

**Appendix 2. Protocol**

**Irrigation Techniques in Spine Surgery**

**-**

**Systematic Review and Meta-Analysis Protocol**

| Title of Review | Irrigation Techniques Used in Spine Surgery for Surgical Site Infection Prophylaxis: A Systematic Review and Meta-Analysis |
| --- | --- |
| First Reviewer | Kabir A. Torres, MSCR |
| Team of Reviewers | Elliot Konrade, MD  Jacob Birlingmair, MD |
| Supervisor/Project PI | Brandon B. Carlson, MD, MPH |
| Support | Mauro Costa M Tavares Junior, MD |
| Clinical Portfolio Group | Joshua T. Bunch, MD  Douglas Burton, MD  R. Sean Jackson, MD |

| **1. Background to Review** |
| --- |
| *Surgical site infections (SSI) are the most common complication after spinal surgery and are associated with greater morbidity, mortality, length of hospital stays and poorer long-term outcomes.^1^ It has been cited that the overall incidence of spinal SSI is 4%^2^, with higher rates seen in implant-related surgery. These higher rates extend up to 9.4% for instrumented spinal surgery for traumatic fractures and 19.2% in pediatric deformity surgery.^3,4^ Previous evidence showed that SSI accounted for 45.6% of readmissions among metastatic tumor patients.^5^ Surgical invasiveness increases the risks for infection. As an example, Fei et al. reported infection rates of 1% for a discectomy or laminectomy compared to 2-5% infection rates in spinal fusion cases.^6^ Another study showed that the anterior-posterior combined surgical approach is the most commonly infected procedure and adding an implant to a spinal fusion further increases the rate of infection ranging from 2.4 to 8.5%.^7^*  *Another, more recent study, reports SSI rates ranging from 0.2% to 16.7% based on sub-stratified characteristics like patient risk factors, perioperative factors and pathology.^8^ A meta-analysis identified several statistically significant risk factors associated with SSI including: diabetes, prolonged operative time of greater than 3 hours, surgery using a posterior approach, morbid obesity with a BMI greater than 35, and number of intervertebral levels of greater than or equal to seven.^6^ Interestingly, the study reported no statistical significance of smoking as an independent risk factor;^6^ however, smoking and nicotine use are known risk factors for SSI in other surgical populations.^9^ Mitigation of risk factors along with appropriate preoperative, intraoperative and postoperative prophylactic measures are all potential areas for decreasing SSI during spinal surgeries. Therefore, it is vital that strategies aimed toward spinal surgery SSI prevention should be elucidated and implemented.*  *Intrawound decontamination prior to closure has become a unique point of interest for research. Present methods include direct application of antibiotic, most commonly vancomycin in a powdered form, to the surgical site.^10-12^ Intrawound vancomycin has been studied quite extensively in recent years. Intrawound antiseptic irrigation is another method of SSI prophylaxis that is often combined with intrawound vancomycin.^13^ The majority of the research has focused on povidone-iodine though concerns exist regarding its toxicity to bone.^14^ Other types of irrigation solutions have minimal evidence suggesting their benefits or risks. The purpose of this systematic review is to evaluate the available research related to the efficacy of different intraoperative irrigation techniques used in spinal surgery for surgical site infection (SSI) prophylaxis.*  *Aim*  *To examine the range of irrigation solution available for use during spine surgery and their outcomes in surgical site infection prophylaxis generating a meta-analysis.* |

| **2. Specific Objectives** |
| --- |
| 1. *To identify irrigation solutions used in spine surgery for the purpose of preventing subsequent surgical site infection. Identification will be completed through a comprehensive multi-database search performed by a Clinical Librarian.* 2. *To evaluate the efficacy of the above identified irrigation solutions by means of critically analyzing the filtered articles and scrutinizing their statistics. This will serve to both learn if there are gaps in knowledge in this space as well as understand what solution are most used based on the evidence at this time.* 3. *Pool all data to generate a viable meta-analysis.* |

| **3a. Criteria for inclusion in the review** | |
| --- | --- |
| i. Population, or participants and conditions of interest | *People undergoing spine surgery; pediatric or adult; no location restriction* |
| ii. Interventions or exposures | *People who has undergone spine surgery and been treated with a prophylactic irrigation of the surgical site* |
| iii. Comparisons or control groups | *People treated with a prophylactic irrigation solution compared to those either with not irrigation or with another irrigation solution* |
| iv. Outcomes of interest | *Surgical site infection* |
| v. Setting | *Hospital admissions/secondary care* |
| vi. Study designs | *Randomized clinical trials, prospective or retrospective cohort studies* |

| **3b. Criteria for exclusion in the review** |
| --- |
| *Studies that do not relate to spine surgery and the use of an irrigation solution for the prophylaxis of surgical site infections.* |

Additional information about inclusion and exclusion criteria:

The inclusion criteria: (1) spine surgeries with intraoperative use of any wound irrigation technique, (2) comparison groups with a different intraoperative irrigation technique or no irrigation, (3) SSI identified with bacterial cultures or clinically in the postoperative period, (4) reported SSI rates. Only articles in English will be considered.

Exclusion criteria: (1) articles with no human subjects, (2) reviews, meta-analyses and case control studies and (3) lack of details about SSI identification or rates.

| **4. Search Methods** | |
| --- | --- |
| Electronic Databases | *Embase*  *OVID Medline*  *Web of Science*  *Cochrane* |
| Other methods used | *N/A* |
| Journals Hands Searched | *N/A* |

| **5. Methods of Review** | |
| --- | --- |
| Details of methods | *Two main reviewers, Kabir and Elliot with Jacob to resolve any conflicts. Rayyan QCRI application will be used to streamline review process.* |
| Quality Assessment | *Protocol will define the method of literature critique/ appraisal use, and will use STROBE tool for relevant content and methodology used in the each of the papers to be reviewed.*  *The prediction model risk of bias assessment tool (PROBAST) will be used for assessing the risk of bias and applicability* |
| Data Extraction | *This will be completed in a Word and Excel document*  *EndNote will be used to keep track of references* |
| Narrative Synthesis | *Narrative synthesis will be done alongside any meta-analysis and will be carried out using a framework which consists of four elements;*  *1. Developing a theory of how the intervention works, why and for whom*  *2. Developing a preliminary synthesis of findings of included studies*  *3. Exploring relationships within and between studies*  *4. Assessing the robustness of the synthesis* |
| Meta-Analysis | *Will be performed using Cochrane Database’s Review Manager software to generate forest and funnel plots.* |
| Grading Evidence | *Planned at the final evaluation* |

Additional information:

DATA EXTRACTION AND OUTCOME MEASURES

For the meta-analysis, only studies related to povidone-iodine will be analyzed to avoid increasing the heterogeneity and maintain high quality of evidence. Sub-analysis will be performed in the povidone-iodine group including only RCT and prospective cohort studies to further increase the quality of evidence. The following variables will be collected in a standardized Excel form: author list, publication years, sample sizes, irrigation technique with control group(s), rates of SSIs (superficial vs. deep when available). Exposure of interest is intraoperative irrigation technique to the surgical wound in spine surgeries. The primary outcome is the rate of SSIs reported in the irrigation technique and control groups. The accuracy of the data extraction will be confirmed independently by 2 coauthors.

DATA SYNTHESES

Descriptive statistics of the control versus experimental groups will be combined and obtained using Microsoft Excel 365 (Microsoft Inc, Seattle, USA). Data will be tested for normally distribution, if non normal distribution has been found, the chi-square statistics will be performed between pooled data to identify any significant difference in observed and expected frequencies of SSIs. Pearson’s chi-square, risk ratio, 95% confidence intervals (CI), and p values will be compiled. P<0.05 is the standard of significance.

HETEROGENEITY AND PUBLICATION BIAS

We will assessed the outcome variables’ heterogeneity using the Q statistic, Tau^2^, I^2^ index and p value under a random-effects model on a forest plot. We will use the Cochrane Database’s Review Manager software to generate forest and funnel plots. A p value for the Q-statistic less than 0.1 suggest that significant heterogeneity exists in the data. Additionally, the I^2^  (range: 0%-100%) value establishes a quantitative degree of variation among included studies. The prediction model risk of bias assessment tool (PROBAST) will be used for assessing the risk of bias and applicability by two reviewers working together.

| **6. Presentation of Results** | |
| --- | --- |
| Additional materials | *Flow chart of whole process*  *Search terms used*  *Protocol*  *Data extraction form and tables*  *Figures* |
| Outputs form Review | *x1 paper in high spine journal*  *Conference presentation*  *Presentation to KU Orthopaedic Department* |

| **7. Timeline for Review** | |
| --- | --- |
| Protocol | *2 weeks* |
| Literature Searching | *1 month* |
| Quality Appraisal | *2 weeks* |
| Data Extraction | *1 month* |
| Synthesis | *1 month* |
| Writing Up | *1 month* |

*The present study was not registered at International Prospective Register of Systematic Reviews (PROSPERO).
